# Adherence with metreleptin therapy and health self-perception in patients with lipodystrophic syndromes

**DOI:** 10.1186/s13023-019-1141-2

**Published:** 2019-07-12

**Authors:** Camille Vatier, Dina Kalbasi, Marie-Christine Vantyghem, Olivier Lascols, Isabelle Jéru, Anne Daguenel, Jean-François Gautier, Marion Buyse, Corinne Vigouroux

**Affiliations:** 1 Sorbonne Université, Inserm UMR_S938, Saint-Antoine Research Centre, Institute of Cardiometabolism and Nutrition (ICAN), Paris, France; 2 Endocrinology, Diabetology and Reproductive Endocrinology Department, National Reference Center for Rare Diseases of Insulin Secretion and Insulin Sensitivity, AP-HP, Saint-Antoine Hospital, 184, rue du Faubourg Saint-Antoine, 75571, 12 Paris Cedex, France; 3 Pharmacy Department, AP-HP, Saint-Antoine Hospital, Paris, France; 4 CHU Lille Department of Diabetology, Endocrinology and Metabolism, Lille University Hospital and Inserm U1190, European Genomic Institute for Diabetes, Lille, France; 5 Biology and Molecular Genetics Department, AP-HP, Saint-Antoine Hospital, Paris, France; 6 Diabetology and Endocrinology Department, AP-HP, Lariboisière-Saint-Louis Hospital Group, Paris, France; 7 Cordeliers Research Centre, Paris-Diderot Paris-7 University, Inserm, UMR_S1138, Paris, France

**Keywords:** Lipodystrophic syndromes, Metreleptin therapy, Health self-perception, Adherence, Social interactions

## Abstract

**Background:**

Although metreleptin replacement therapy was shown to improve metabolic alterations in lipodystrophic syndromes, patients’ adherence and satisfaction with treatment have never been evaluated. The 20 patients with lipodystrophic syndromes participating in the French compassionate program of metreleptin therapy filled in a self-questionnaire including an Adherence Evaluation Test, the Treatment Satisfaction Questionnaire for Medication (TSQM®-vII), and items about physical appearance.

**Results:**

15 patients were women, median age was 32.5 years (IQT 25–75 (16.2;49.5), 18 had diabetes. Adherence with metreleptin (one daily subcutaneous injection) was poor in 25%, excellent in 25% and acceptable in 50% of patients. On a 0-to-100 scale, patients’ satisfaction scores reached 66.7 (52.1;81.2) for effectiveness, 55.6 (44.4;66.7) for ease/comfort of use, and 83.3 (52.1;83.3) for global satisfaction with metreleptin therapy. Self-reported side effects were frequent injection site reactions 100 (79.2;100). Satisfaction scores did not differ in patients with partial (*n* = 10) or generalized (*n* = 10) lipodystrophic syndromes, did not correlate with metabolic improvement, but were significantly higher in compliant patients with fewer side effects. Morphological appearance was reported improved under metreleptin therapy in 13 among 17 patients.

**Conclusions:**

Metreleptin increases health self-perception and decreases morphotype-associated stigmatization in most patients with lipodystrophic syndromes, but poor comfort of use and local side effects weaken adherence.

## Background

Chronic conditions may have major negative impact on individuals’ lifes. To assess patients’ adherence, self-experience with treatment and health-related quality of life has become an integral part of follow-up in chronic diseases, providing important decision-making criteria for therapeutic management. Lipodystrophic syndromes (LD) are chronic diseases of acquired or genetic origin characterized by body fat loss and metabolic complications associated with insulin resistance, i.e. glucose tolerance abnormalities, hypertriglyceridemia, liver steatosis, and ovarian hyperandrogenism in females [1]. Lipoatrophy can be generalized, or partial as in Familial Partial Lipodystrophy (FPLD), where peripheral subcutaneous lipoatrophy contrasts with cervicofacial fat accumulation, resulting in a cushingoïd appearance [1].

Lipodystrophic morphological changes could be dramatically stigmatizing, although few studies evaluated the psychological consequence of these rare diseases [2–7].

Leptin deficiency due to fat loss contributes to the metabolic complications of LD. Metreleptin replacement therapy was shown to increase insulin sensitivity and insulin secretion, and to decrease hyperglycemia, hypertriglyceridemia, liver steatosis, and reproductive abnormalities associated with LD. Metreleptin is currently approved in the US, Japan and Europe to treat rare forms of severe LD. However, its effect on health-self perception is poorly known, although a subjective improvement was reported in three patients with generalized LD under metreleptin [8].

As the French Reference Center for Rare Diseases of Insulin Secretion and Insulin Sensitivity, we evaluated the patients’ self-experience regarding adherence and satisfaction with treatment, including physical appearance and social interactions, in 20 patients with LD included in the national compassionate program of metreleptin therapy.

## Results

### Characteristics of the patients and metabolic changes under metreleptin therapy

All the 20 patients treated by metreleptin in 2015 through the French LD compassionate program agreed to participate in the study.

Patients were 13 to 71 years old (median (25;75 IQT): 35 (22.0;51.7). Fifteen of them (75%) were women. Ten patients (50%) had generalized LD due to *AGPAT2* (CGL1) or *BSCL2* (CGL2) biallelic pathogenic variants or *LMNA* Asp47Tyr heterozygous variant (5 patients with CGL1 and 2 with CGL2, and 1 with progeroid laminopathy respectively), or associated with auto-immune disorders (*n* = 2). Ten patients had partial lipodystrophy, due to *LMNA* Arg482Trp (FPLD2, *n* = 7) or *PPARG* (FPLD3, n = 2) heterozygous variants, or of unknown origin (*n* = 1) (Table 1). With the exception of two brothers with CGL2 treated with metreleptin since childhood for hypertriglyceridemia and insulin resistance for a total of 108 months, all patients had diabetes and duration of metreleptin therapy ranged from 12 to 61 months. Metreleptin was self-administrated by the patients at doses ranging from 0.04 to 0.19 mg/kg/d (median 0.11 mg/kg/d) in one subcutaneous injection per day.

**Table 1:** Characteristics of patients at the time of evaluation of metreleptin therapy

Patients	Age (years)	Sex (M/F)	Disease (gene mutation)	Baseline serum leptin (ng/ml)	Baseline/Delta BMI (kg/m^2^)	Baseline/Delta Tg (mmol/l)	Baseline/Delta HbA1c (%, pt)	Duration of metreleptin therapy (months)	Treatment adherence	Effectiveness (%)	Side effects (%)	Ease and comfort of use (%)	Global satisfaction (%)	Changes in physical appearance	Changes in social interactions
1	64	F	FPLD2	0.59	22.8	1.93	7.7	60	A	66.7	100	66.7	66.7	+	+
					−2.4	−0.04	− 0.5								
2	41	F	FPLD2	1.43	25.7	3.32	7.7	60	P	58.3	41.7	33.3	50	–	+
					−2.9	−0.35	0.1								
3	29	F	CGL1 (HMZ *AGPAT2* p.L165-Q196del)	0.13	26.8	10.41	8.7	61	E	83.3	100	83.3	83.3	++	++
					−0.8	−6.73	−1.9								
4	22	F	CGL1 (HMZ *AGPAT2* p.Q196fsX228)	1.73	20.3	1.79	7.6	54	P	66.7	50	44.4	58.3	++	0
					−1.7	−0.51	−2.5								
5	27	F	FPLD (unknown origin)	4.5	21.4	32.70	10.6	55	A	58.3	91.7	55.6	66.7	++	++
					0.3	−12.72	− 0.7								
6	19	M	AGL	0.15	23.1	12.75	11.9	60	P	41.7	50	33.3	33.3	NC	+
					2.0	−11.46	−4.0								
7	56	F	CGL1 (HMZ *AGPAT2* p.K216X)	0.15	21.5	4.02	8.1	59	E	83.3	91.7	72.2	83.3	–	++
					−3.3	−2.68	0.0								
8	54	F	FPLD2	4.12	24.5	1.76	8.5	48	E	83.3	100	66.7	83.3	++	+
					−2.7	0.75	−0.8								
9	45	F	FPLD2	3.44	23.0	2.58	6.9	48	E	83.3	100	66.7	83.3	++	++
					−3.7	0.36	0.3								
10	22	F	FPLD2	5.64	25.0	9.87	12.1	48	A	75	100	66.7	83.3	++	++
					−1.5	−1.93	−3.3								
11	53	F	FPLD2	3.60	23.2	1.5	7.1	48	A	66.7	100	66.7	83.3	+	+
					−1.9	1.03	−0.7								
12	19	F	Progeroid LD (HTZ *LMNA* p.D47Y)	1.12	17.4	5.0	10.4	48	P	50	75	44.4	50	+	0
					−1.4	0.26	−2.9								
13	39	F	FPLD2	3.92	25.1	2.82	7.1	48	A	66.7	100	50	83.3	++	++
					−1.9	−1.36	−1.1								
14	47	M	FPLD3 (HTZ *PPARG* p.L339X)	4.20	29.0	9.26	10.8	42	A	41.7	91.7	50	58.3	NC	+
					−1.8	2.87	−1.5								
15	31	F	CGL1 (HMZ *AGPAT2* p.Q196fsX228)	2.18	23.7	1.00	8.7	36	P	50	100	44.4	50	– –	+
					−1.0	−0.03	- 0.3								
16	13	F	AGL	2.8	18.7	20.46	12.2	22	A	33.3	41.7	33.3	50	–	– –
					−0.1	−15.13	−3								
17	71	M	CGL1 (HMZ *AGPAT2* p.E172K)	0.34	20.0	5.29	8.3	18	A	75	100	55.6	83.3	NC	++
					0.0	−3.29	−1.8								
18	48	F	FPLD3 (HTZ *PPARG* p.L339X)	8.4	31.1	26.67	8.0	12	A	91.7	100	55.6	100	++	++
					−0.7	−23.52	− 0.3								
19	18	M	CGL2 (HMZ *BSCL2* p.R138X)	3.4	19.9	2.74	5.5	108	A	75	100	55.6	83.3	++	++
					0.7	−0.19	−0.3								
20	22	M	CGL2 (HMZ *BSCL2* p.R138X)	0.8	25.3	5.25	5.3	108	E	75	100	55.6	91.7	++	++
					−0.8	−0.72	−0.2								
Median (IQT 25;75)	35.0 (22.0–51.7)			2.5 (0.6;4.1)	23.4 (20.6;25.3)	4.5 (2.4;10.3)	8.2 (7.2;10.6)	51.0 (43.5;60)		66.7 (52.1;81.2)	100 (79.1;100)	55.7 (44.4;66.7)	83.3 (52.1;83.3)		
					−1.4 (− 0.2;-2.3)	− 0.6 (− 0.2;-5.9)	− 0.7 (− 0.2;-1.9)								

Baseline values were obtained at the time of initiation of metreleptin therapy. Delta relates to the difference between pretherapeutic and current levels of HbA1c and Tg (triglycerides). *CGL* congenital generalized lipodystrophy, *AGL* acquired generalized lipodystrophy, *HMG* homozygous, *HTZ* heterozygous, FPLD2: HTZ *LMNA* p.R482W, Treatment adherence was assessed as E, excellent; A, acceptable; P, poor and changes as ++ strongly improved, +improved, 0 no effect, − worsened, −- strongly worsened, *NC* the patient did not feel concerned, *SD* standard deviation; IQT, interquartile range 25–75%

At the time of initiation of metreleptin therapy, median (IQT) levels of BMI, HbA1c and serum triglycerides (Tg) were 23.4 kg/m^2^ (20.6;25.3), 8.2% (7.2;10.6) and 4.5 mmol/L (2.4;10.3), respectively. At the time of evaluation, BMI, and HbA1c and Tg had decreased from pre-metreleptin therapy levels by a median of 1.4 kg/m^2^ (–0.2–;2.3), 0.7% (–0.2–;1.9) and 0.6 mmol/L (–0.2;–5.9) respectively (Table 1). However, patient 14 with FPLD3 (*PPARG*-FPLD) had a 2.87 mmol/l increase in Tg from baseline, associated with inappropriate diet at the time of evaluation. Four patients had stopped their insulin therapy under metreleptin (patients 4, 7, 9 and 12).

### Patients’ opinion regarding metreleptin therapy

**Fig. 1:**
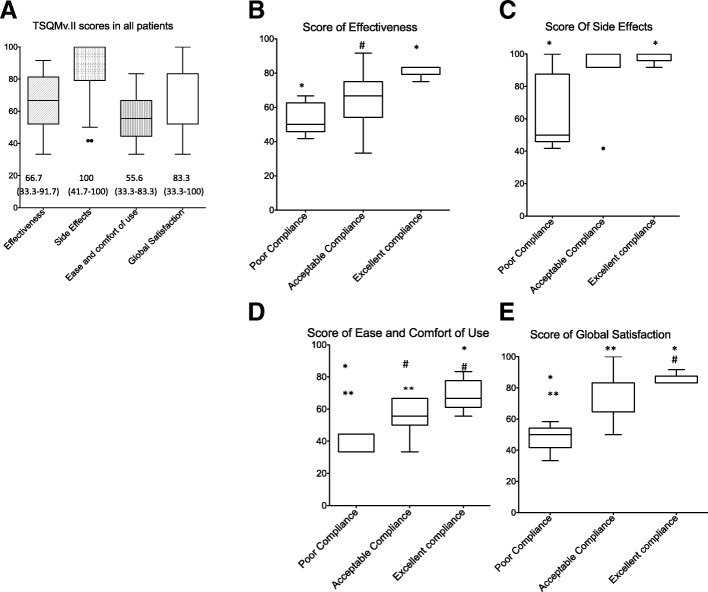
Results of the Treatment Satisfaction Questionnaire for Medication (TSQM®-v.II) in patients with lipodystrophic syndromes treated by metreleptin. Rectangles represent 25th and 75th percentile values with median value depicted inbetween. Whiskers represent the lowest datum still within 1.5 interquartile range of the lower quartile, and the highest datum still within 1.5 interquartile range of the upper quartile (Tukey boxplot). **a**, scores for each item of the TSQMv.II self-questionnaire in all patients, **b**-**e**, scores for each item in the three adherence groups of patients (poor compliance (**p**), *n* = 5; acceptable compliance (**a**), *n* = 10; excellent compliance (**e**), *n* = 5). # *p* < 0.05 between **a** and **e** groups, * *p* < 0.05 between **p** and **e** groups and ** *p* < 0.05 between **p** and **a** groups

The median TSQM®-vII global satisfaction score of the patients was 83.3 (52.1;83.3) on a scale of 0 to 100, and the proportion of patients with a mean TSQM®-vII global satisfaction score greater than or equal to 50 was 95%. Patients rated the effectiveness of metreleptin therapy at 66.7 (52.1;81.2), and its ease and comfort of use at 55.6 (52.1;83.3). The reported side effects (pain, redness and/or skin induration at injection sites) were rated with a high score (100 (79.2;100)). Figure 1a presents the median (range) score for each item. Six patients added a free text comment related to the practical difficulties linked to the daily reconstitution of the product from powder and/or the subcutaneous route of injection in the absence of a pre-prepared device. Neither gender, duration of metreleptin therapy, nor changes in BMI, HbA1c or serum triglycerides from baseline levels at the time of evaluation were related to TSQM®-vII scores.

### Adherence to treatment

Metreleptin therapy adherence was excellent in 5 among the 20 studied patients (25%), acceptable in 10 of them (50%) and poor in 5 of them (25%) (Table 1), as assessed by the Adherence Evaluation Test (AET) (Table 1). Patients’ self-evaluation of metreleptin effectiveness, ease and comfort of use and side effects, as well as their global satisfaction with therapy, evaluated by the TSQM®-vII scores, were significantly related to their adherence to treatment (Fig. 1b-e). Notably, local side effects were highly prevalent in patients with excellent or acceptable adherence (score of 100 (95.8;100) and 100 (91.7;100) respectively), and significantly less prevalent in patients with poor adherence to treatment (score of 50 (45.8;87.8) (Fig. 1c). However, changes in BMI, HbA1c and triglyceride levels from pretherapeutic values were not significantly different among patients classified with poor, acceptable or excellent metreleptin therapy adherence (Table 1).

### Patients’ evaluation of physical appearance and social interactions since the initiation of metreleptin therapy

Physical appearance was experienced as stigmatizing in all patients except three men (one with FPLD and two with generalized LD).

Thirteen patients reported that their physical appearance was improved or strongly improved under metreleptin therapy (5 patients with generalized and 8 with partial LD). In contrast, four patients (3 with generalized and one with partial lipodystrophy), all with a poor metreleptin therapy adherence, reported that it worsened or strongly worsened under treatment (Table 1). In addition to an improvement in facial morphotype, free text comments from 10 patients mentioned a decrease in skin thickening under metreleptin therapy.

Half of patients under metreleptin therapy reported a very positive effect on social interactions (familial, professional and social life). 7 other patients reported a positive effect whereas two reported no effect and one a negative effect on social interactions. The latter patient, 13 years old, with acquired generalized lipodystrophy, was also unsatisfied regarding her physical appearance under treatment (patient 16, Table 1). There was no correlation between social interactions improvement and baseline metabolic parameters or their changes under metreleptin, but initial BMI was significantly higher in the group with very positive or positive effect of metreleptin therapy on social interactions as compared to the group with none or negative effect (24.3 ± 2.9 kg/m^2^, *n* = 17 versus 18.8 ± 1.4 kg/m^2^, *n* = 3).

### Results according to the type of lipodystrophy

At baseline, there was no significant difference regarding metabolic parameters between generalized and partial lipodystrophic patients (HbA1c, Tg, BMI), but, as expected leptinemia was lower in patients with generalized versus partial lipodystrophy (Table 2). Under metreleptin therapy, decrease of BMI was higher in the partial group. Similar decrease in HbA1c and Tg was observed in the two groups of patients (Table 2).

**Table 2:** Baseline characteristics, metabolic changes and patients’ self-evaluation of metreleptin therapy according to the type of lipodystrophy

		Partial LD (*n* = 10)	Generalized LD (*n* = 10)	p
Baseline characteristics	Age (years)	43.0 (33.2;50.0)	16.5 (11.7;34.7)	0.04
	BMI (kg/m^2^)	25.0 (22.9;26.5)	20.9 (19.6;24.1)	0.035
	Leptinemia (ng/mL)	4.0 (2.9;4.8)	1.0 (0.1;2.3)	0.003
	HbA1c (%)	7.8 (7.1;10.6)	8.5 (7.1;10.8)	0.73
	Tg (mmol/L)	3.1 (1.9;14.1)	5.1 (2.7;11.0)	0.97
Metabolic changes	Delta HbA1c (%)	−0.7 (− 0.2; − 1.2)	−1.2 (− 0.1; −2.9)	0.6
	Delta Tg (mmol/L)	− 0.2 (−4.6;0.8)	− 1.7 (− 0.1;-7.9)	0.2
	Delta BMI (kg/m^2^)	−1.9 (− 1.3;-2.7)	− 0.8 (− 1.4; 0.2)	0.035
	Delta number of anti-diabetic medications	0 (− 1;1)	0 (− 3; 1)	0.94
	Delta number of patients under insulin	−1	− 3	
Self-evaluation of treatment	Efficiency	66.7 (58.3;83.3)	70.8 (47.9;77.1)	0.59
	Side effects	100 (91.7;100)	95.8 (50;100)	0.32
	Ease and comfort of use	61.1 (50;66.7)	50.0 (41.7;59.7)	0.30
	Global satisfaction	83.3 (64.6;83.3)	70.8 (50.0;83.3)	0.36
	Changes in physical appearance	6 ++ 2 + 1 NC 1 -	4 ++ 1 + 2 - 1 - - 2 NC	
	Changes in social interactions	4 ++ 6 +	5 ++ 2 + 1 - - 2 no effect	
Adherence	Acceptable or excellent metreleptin adherence (number of patients)	9	6	

*Tg* triglycerides, *LD* lipodystrophy

Patients with acceptable or excellent metreleptin therapy adherence were classified as “compliant” whereas patients with poor metreleptin therapy adherence were considered as “non compliant”; Self-evaluation of the effect of metreleptin therapy was ++ significantly improved, +improved, 0 neutral, − worsened, or -- significantly worsened; NC not concerned

TSQM®-vII scores were not significantly different in patients with partial or generalized lipodystrophy, nor was adherence to metreleptin therapy, which was evaluated as poor in four patients with generalized lipodystrophy and one patient with partial lipodystrophy.

**Fig. 2:**
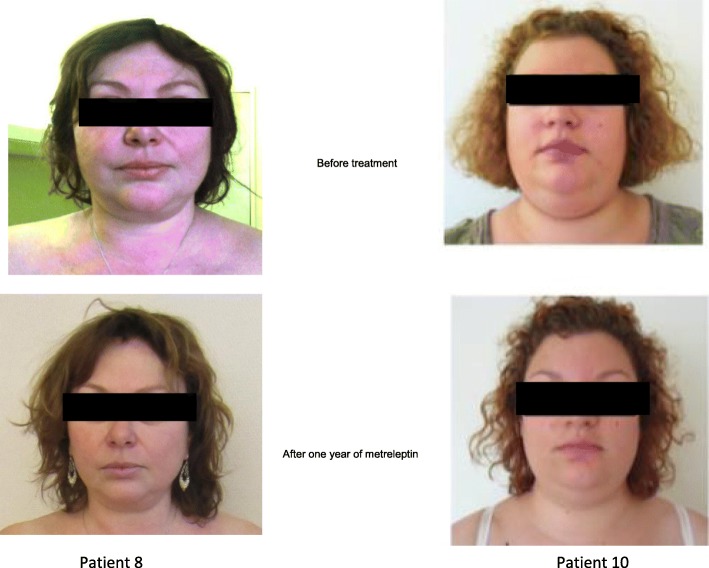
Morphological facial changes after one-year metreleptin therapy in two patients with FPLD2

All women with partial lipodystrophy (*n* = 9) viewed their physical appearance as stigmatizing, and all of them, except one poorly compliant patient, reported that it improved (*n* = 2) or strongly improved (*n* = 6) under metreleptin therapy (Table 2). Two men and 6 women among 10 patients with generalized LD experienced their morphological appearance as stigmatizing. These two men, and two of the 6 women reported that metreleptin strongly improved their morphological phenotype (Tables 1 and 2). The decrease in faciocervical fat accumulation under metreleptin therapy in patients with FPLD2 is illustrated in Fig. 2.

## Discussion

We have assessed the patients’ adherence and satisfaction with metreleptin therapy, as well as self-perception of physical appearance and social interactions, in all the 20 patients with partial and generalized LD included in the French metreleptin compassionate program and treated for more than 1 year at the time of the study. In lipodystrophic syndromes, health self-perception may be impaired not only by the metabolic complications, but also by the morphological stigmatizing features associated with the different forms of the disease. Indeed, besides specific dysmorphic traits which characterize rare forms of LD, lipoatrophy, cervicofacial fat accumulation and/or acromegaloid features could affect self-esteem and social interactions in patients with LD, as suggested by previous studies, which however only systematically investigated patients with HIV-related forms of lipodystrophy [2–6]. Although metreleptin replacement therapy was shown to improve metabolic alterations in LD, justifying its approval in the US, Japan, and recently in Europe in the severe forms of the disease, adherence to therapy and its effect on health-related self-perception has not been evaluated.

Using a validated self-questionnaire, we show that patients were generally satisfied with this treatment. This positive perception of metreleptin therapy was not significantly influenced by gender, type of lipodystrophy, age, previous duration or objective metabolic efficacy of therapy. However, adherence with metreleptin therapy was evaluated as poor in 25% of the patients. Sub-optimal ease and comfort of use of metreleptin may result, at least in part, from the daily reconstitution from powder and from the subcutaneous injection route of administration, as suggested by patients’ free text comments. An easier system of use as an auto-injector, a product ready to use, and/or a long-lasting molecule allowing a weekly injection could improve adherence to treatment. In addition, the high prevalence of local side effects probably due, at least partly, to painful injections in lipoatrophic areas. However, although we did not observe any correlation between objective metabolic improvement and adherence to treatment, the patients’ self-perception of metreleptin effectiveness and comfort of use, and their global satisfaction with therapy, were strongly related to adherence. Accordingly, a systematic review of illness perceptions in mental health found that positive perceptions of treatment were linked to better adherence [9].

This study also shows that the phenotype associated with LD, of generalized or partial type, is perceived as stigmatizing by the majority of patients. All the women from this study reported to suffer from their physical appearance. Moreover, as a consequence, social interactions were experienced as difficult by a majority of patients. This patients’ experience should be taken into account for the management of the disease. Improvement of physical appearance under metreleptin was reported by 76.5% of patients. It was the case for all women affected with partial lipodystrophy, except one with poor adherence with therapy, who reported a decrease in faciocervical fat accumulation. This result is in accordance with a study from Miehle et al. showing that metreleptin reduces facial soft tissue volume in lipodystrophy [10]. The majority of patients with generalized LD also mentioned that their physical appearance had improved under metreleptin therapy. From the patients’ feedback, it is likely that this results from a major improvement in insulin resistance-linked *acanthosis nigricans*, which was frequently broadly extended throughout the body at baseline, with increased thickness of the skin, and participated in the morphological discomfort of the patients. Whatever the reason however, metreleptin therapy provides an improvement in social interactions as self-assessed by the majority of the patients. Finally, the perception of illness, which refers to individual experience, is known to influence the outcome of a variety of diseases, including cancer [11], cardiovascular diseases [12], chronic fatigue syndrome [13], eating disorders [14] and diabetes [15, 16]. The positive perception of metreleptin therapy by patients with LD could therefore participate in its effects on metabolic parameters.

Limitations of the study include its cross-sectional, exploratory and descriptive design, the limited number of studied patients, and the lack of evaluation of health-self perception before metreleptin therapy. A standardized questionnaire collecting Patient-Reported Outcomes in each country with access to metreleptin compassionate therapeutic programs would have allowed to draw a more complete and reliable picture of patients’ self-perceived health during treatment. This would have highlighted the patients’ expectations for a better ease and comfort of use of the medication. However the context of compassionate programs itself might influence the results due to psychological bias. The commitment of patients to use the product, as well as their expectations regarding its efficiency might be different in real life. Further information is thus needed regarding long-term patients’ adherence and perception of metreleptin therapy. This would usefully form a part of a multicentric post-marketing study of metreleptin therapy in patients with lipodystrophy.

## Conclusions

However, this study highlights that besides its benefits on lipodystrophy-related metabolic complications, metreleptin therapy is able to improve the patients’ health self-perception and to decrease their morphotype-associated stigmatization.

## Methods

### Patients and study

All 20 patients, without HIV infection, with genetic or acquired, partial or generalized lipodystrophy, diabetes and low serum leptin levels (fasting leptin ≤8.5 ng/ml), included in a compassionate program of metreleptin therapy approved by the National French Health Agency, were proposed during year 2015, as part of a previous study evaluating metabolic results of the program [17], to fill in a 30-min self-questionnaire evaluating adherence and satisfaction with metreleptin. This assessment was only made once. All the patients accepted to enter this exploratory descriptive study. Metreleptin was added to the regular treatment of the patients since 12 months to 108 months (median 51 months). A marketing authorization for metreleptin was previously obtained in the US and in Japan. In the European Union, metreleptin was available through compassionate programs before its authorization as Myalepta®, Aegerion Pharmaceuticals, on 2018, July 29th. For the French metreleptin compassionate program, patients were given by the hospital pharmacist 90 vials of 11.3 mg of metreleptin powder (to be kept refrigerated), with an equal number of vials of sterile water, 3 ml-syringes with needles for reconstitution, and 2 ml-syringes with needles for subcutaneous injection, every 3 months. Patients had to daily reconstitute a 5 mg/mL metreleptin solution (using 2.2 mL of water for injection for a vial of powder), to draw the amount prescribed and and inject it subcutaneously in the abdomen. Metreleptin and additional treatments were adapted to the metabolic results, which were collected during the medical follow-up, every three to 6 months. All the patients signed an informed consent for this study which was approved by our local ethics committee [17].

### Design and interpretation of the self-questionnaire

The self-questionnaire included an Adherence Evaluation Test (AET), derived from the Morisky-Green Medication Adherence Questionnaire validated in French [18] and the Treatment Satisfaction Questionnaire for Medication (TSQM®-vII) [19]. Six AET items with dichotomous responses (yes or no), allowed to classified patients with “excellent” (score  =  0), “adequate” (score  =  1 or 2) or “poor” adherence (score = 3 to 6) to metreleptin therapy. Eleven TSQM®-vII items, scored on a 7-point Likert scale, were dedicated to the patients’ self-perception of effectiveness (i.e. ability of the medication to treat the condition and relieve symptoms), side effects, ease and comfort of use of metreleptin therapy, and evaluated the patients’ global satisfaction with the treatment (relative weight of advantage vs disadvantages). Each TSQM®-vII score was transformed into a 0-to-100 rating. Patients were also asked to describe the potential side effects of metreleptin. Finally, we added specific questions assessing the patients’ self-opinion regarding changes in physical appearance and social interactions since the initiation of metreleptin therapy (“Today are you satisfied with changes regarding your physical appearance – or social interactions – since the beginning of the treatment? ”, with 5 possible answers: “yes totally, yes a bit, no effect, not satisfied and not satisfied at all”), as well as a non-directed free text comment.

### Statistics

Statistical analyses were performed using GraphPad PRISM (GraphPad Software, Inc., CA, USA) statistical software. Descriptive statistics included numbers (%) for categorical variables and, for quantitative variables, median (IQT 25;75). We used the Fisher exact test to compare categorical variables and the non-parametric Mann Whitney U test to compare quantitative variables. Correlations of different measures of metabolic parameters with type of lipodystrophy or adherence or TSQM®-vII were evaluated using Spearman’s rank correlation test or linear regression analysis. *P* values < 0.05 were considered significant.

## Data Availability

C.Vatier, C. Vigouroux, store all data which are available.
